# Development and validation of the FAAP model for prognostic stratification in HCC patients treated with TACE, sintilimab plus bevacizumab: a multicenter study

**DOI:** 10.3389/fimmu.2025.1692632

**Published:** 2025-11-25

**Authors:** Liying Ren, Dongbo Chen, Xue Zhang, Shaoping She, Ran Fei, Xu Cong, Shaowei Mu, Yuchen Zhou, Jie Gao, Weijia Liao, Hongsong Chen

**Affiliations:** 1 Peking University People’s Hospital, Peking University Hepatology Institute, Infectious Disease and Hepatology Center of Peking University People’s Hospital, Beijing Key Laboratory of Hepatitis C and Immunotherapy for Liver Diseases, Beijing International Cooperation Base for Science and Technology on NAFLD Diagnosis, Beijing, China; 2 Department of Hepatobiliary Surgery, Peking University People’s Hospital, Beijing, China; 3 Department of General Surgery, Integrated Hospital of Traditional Chinese Medicine, Southern Medical University, Guangzhou, China; 4 Department of Hepatobiliary Surgery, Nanfang Hospital, Southern Medical University, Guangzhou, China; 5 Laboratory of Hepatobiliary and Pancreatic Surgery, Affiliated Hospital of Guilin Medical University China, Guilin, Guangxi, China; 6 Peking University Third Hospital, Beijing, China

**Keywords:** hepatocellular carcinoma, immunotherapy, transcatheter arterial chemoembolization, prognostic model, FAAP score

## Abstract

**Introduction:**

Although the transcatheter arterial chemoembolization (TACE) combined with sintilimab and bevacizumab improves outcomes in unresectable HCC (uHCC), predictive tools are lacking. This study developed and validated a prognostic model for triple therapy efficacy.

**Methods:**

A multicenter study enrolled uHCC patients receiving TACE-sintilimab-bevacizumab. Overall survival (OS) was the primary endpoint; a Cox model was developed and validated.

**Results:**

This study enrolled 147 patients (training cohort: n = 92; validation cohort: n = 55). The optimal cutoff value for the fibrin degradation product-to-cholinesterase ratio*1000 (FCR) was determined as 0.8. Univariate and multivariate Cox regression analyses identified FCR, AST, AFP, and PVTT as independent OS predictors. These variables were integrated to establish the FAAP scoring system, which demonstrated robust discriminative performance with AUC of 0.804 (95% CI: 0.703-0.893) and 0.799 (95% CI: 0.67-0.911) in the training and validation cohorts, respectively. Patients were stratified into three risk groups based on FAAP scores: low (FAAP < 0.7), intermediate (0.7 ≤ FAAP < 2.2), and high (FAAP ≥ 2.2). Kaplan-Meier analyses revealed significant prognostic stratification for both OS and progression-free survival (PFS) across groups. Subgroup analyses confirmed the prognostic relevance of FAAP scores in key clinical subsets, including age, gender, extrahepatic metastasis status, viral hepatitis etiology, PVTT presence, and Child-Pugh stage.

**Conclusions:**

The FAAP scoring system effectively predicted survival outcomes in HCC patients receiving TACE-sintilimab-bevacizumab therapy, which suggests its clinical utility for prognostic prediction. Further large prospective studies are required for external validation.

## 1 Introduction

Hepatocellular carcinoma (HCC) is one of the leading causes of cancer-related death worldwide (1), surgery and liver transplantation are considered curative treatments for HCC. However, due to the insidious onset of HCC, more than 60% of cases diagnosed too advanced for radical resection (2, 3). Patients with unresectable HCC (uHCC) face an unfavorable prognosis under conventional therapies, highlighting the critical need for innovative treatment strategies (4, 5).

The advent of immune checkpoint inhibitors (ICIs) targeting programmed cell death protein-1 (PD-1) and anti-vascular endothelial growth factor (VEGF) agents has revolutionized systemic therapy for uHCC (6–9). In China, the combination of sintilimab and bevacizumab emerged as a first-line treatment following the landmark ORIENT-32 trial, which demonstrated superior progression-free survival (PFS) and overall survival (OS) compared to sorafenib (10, 11). Transcatheter arterial chemoembolization (TACE) provides a targeted locoregional approach by directly delivering chemotherapeutics and embolizing tumor-feeding vessels to effectively reduce tumor burden, which was widely used in Asian countries (12–16). CHANCE2201 studies report encouraging outcomes for TACE combined with ICIs and anti-VEGF triple therapy, triple therapy prolonged the median survival by 6.7 months compared with the control (17). However, how to predict the prognostic risk remain the huge research gap right now. To our knowledge, there are currently no prognostic models for uHCC patients receiving TACE, sintilimab and bevacizumab treatment.

To address this gap, we developed and validated the FAAP prognostic scoring system—a novel composite model integrating fibrin degradation product-to-cholinesterase ratio*1000 (FCR), aspartate aminotransferase (AST), AFP, and portal vein tumor thrombosis (PVTT) to predict survival in uHCC patients undergoing TACE-sintilimab-bevacizumab therapy. This tool holds immediate clinical relevance for optimizing patient selection, guiding adaptive therapeutic escalation, and standardizing efficacy evaluation in trials exploring TACE-immunotherapy-antiangiogenesis combinations.

## 2 Methods

### 2.1 Patients

This is a multicenter retrospective study enrolled patients with uHCC who received TACE combined with sintilimab (anti-PD-1) plus bevacizumab (anti-VEGF) from Peking University People’s Hospital (n = 48, part of training cohort), Nanfang Hospital (n = 44, part of training cohort) and Affiliated Hospital of Guilin Medical University (n = 55, validation cohort) between April 2021 and December 2023. Follow-up was closed on April 2025. Patients without the event were censored at the date of last contact or at end date, whichever occurred first. In accordance with the requirements of the Ethics Committee (18), the study protocol was approved by the Institutional Review Board (IRB) of each center and conducted in accordance with the principles of the Declaration of Helsinki. Written informed consent was obtained from all participants prior to initiating the combined therapy.

### 2.2 Diagnostic criteria and exclusion criteria

HCC diagnosis was established using non-invasive imaging criteria per the American Association for the Study of Liver Diseases (AASLD) and European Association for the Study of the Liver (EASL) guidelines (19, 20). Tumor unresectability was defined by either advanced disease stage (e.g., multifocal lesions, vascular invasion, or extrahepatic spread) or insufficient post-resection liver remnant volume (< 40% for cirrhotic patients and < 30% for non-cirrhotic patients). Inclusion criteria were: 1) Age > 18 years; 2) Patients with unresectable HCC (uHCC) receiving first-line therapy with TACE, sintilimab and bevacizumab; 3) At least one measurable lesion per modified Response Evaluation Criteria in Solid Tumors (mRECIST) (21). Exclusion criteria included: 1) Presence of concurrent malignancies; 2) Eastern Cooperative Oncology Group Performance Status (ECOG-PS) score > 1; 3) Child-Pugh C; 4) Prior locoregional or systemic therapy for HCC; 5) Active autoimmune diseases or severe hematological disorders; 6) Missing follow-up data.

### 2.3 The combination therapy

#### 2.3.1 TACE

The conventional transarterial chemoembolization (C-TACE) procedures were performed by experienced interventional radiologists according to Chinese guidelines (22). Under local anesthesia, femoral artery access was obtained via the Seldinger technique, followed by selective angiography of the celiac trunk or hepatic artery to delineate vascular anatomy, tumor characteristics (number, size, location, vascular staining), and portal vein patency. A coaxial microcatheter (2.2-2.8F) was advanced superselectively into tumor-feeding segmental or subsegmental arterial branches. After angiographic confirmation of the target vessel, an emulsion of iodized oil (5–30 mL) and chemotherapeutic agents (e.g., Epirubicin 40–60 mg) was injected under fluoroscopic guidance until flow stasis or retrograde filling of peritumoral portal branches was observed. Subsequent embolization with polyvinyl alcohol embolization microspheres was performed to achieve complete occlusion of tumor-feeding arteries. Chemotherapy dosages were individualized based on body surface area, tumor burden, and functional status, while iodized oil volume (typically ≤ 20 mL per session) was adjusted according to tumor size, vascularity, and procedural objectives. Dynamic fluoroscopic imaging documenting iodized oil deposition patterns and superselective angiographic sequences were systematically archived for procedural validation and follow-up analysis. TACE was repeated when clinical benefit was anticipated, viable tumor or intrahepatic progression with acceptable liver function status, and withheld if liver function worsened, no treatable arterial target was available, or prespecified TACE-untreatable-progression criteria were met.

#### 2.3.2 Sintilimab and bevacizumab administration

Sintilimab (200 mg) and bevacizumab (15 mg/kg) were administered via intravenous infusion every 3 weeks. The combination therapy cycle was maintained until disease progression or intolerable toxicities. Safety was assessed via treatment related adverse events (TRAEs), which were monitored and recorded in accordance with the Common Terminology Criteria for Adverse Events Version 5.0.

### 2.4 Follow-up surveillance

Patients underwent clinical monitoring including laboratory testing and radiological examinations by contrast-enhanced MRI or CT every 4–8 weeks. PVTT was determined on contrast-enhanced CT/MRI. All baseline scans (within 4 weeks before treatment) were independently reviewed by two abdominal radiologists; discrepancies were resolved by consensus. PVTT was defined as solid intraluminal lesions within the portal vein that demonstrate partial arterial-phase enhancement and portal-phase filling defects on contrast-enhanced imaging. Treatment response was assessed by two independent radiologists by mRECIST criteria. The primary outcome was OS in this study, defined as the time span from the date when patients met the eligibility criteria and initiated the initial combination therapy until the occurrence of death from any cause, data censoring, or the end of follow-up, whichever occurred first. The secondary outcomes included PFS, defined as the time from treatment initiation until radiological progression or death from any cause. Objective response rate (ORR), incorporating complete response (CR, disappearance of arterial-enhancing targets) and partial response (PR, ≥ 30% reduction in enhancing lesion diameter).

### 2.5 Modeling and validation

Continuous clinical variables were dichotomized using established reference ranges or clinically validated cutoffs. Candidate predictors achieving significance (p < 0.05) in univariate Cox regression analyses advanced to multivariate modeling. The FAAP (Fibrin degradation product-to-cholinesterase ratio, Aspartate aminotransferase, Alpha-fetoprotein, and Portal vein tumor thrombosis) scoring system was derived from multivariate Cox regression. Each predictor was dichotomized, and regression coefficients (β) were used as weights to calculate the FAAP score as: FAAP = 0.891 × FCR (0/1) + 0.746 × AST (0/1) + 0.526 × AFP (0/1) + 0.528 × PVTT (0/1). Patients were stratified into risk groups according to X-tile determined cutoffs., weighting variables by their β coefficients. The model’s discriminative capacity was assessed in both training and validation cohorts using time-dependent receiver operating characteristic (ROC) curves, with optimal cutoffs determined via Youden’s index. Risk stratification based on FAAP scores enabled Kaplan-Meier survival curve generation, with between-strata comparisons performed through log-rank testing.

### 2.6 Statistical analysis

Categorical baseline characteristics were presented as frequencies with percentages. Between-group comparisons in training and validation cohorts were performed using Fisher’s exact test or Pearson’s chi-square test. Survival analyses were conducted through univariate and multivariate Cox proportional hazards regression models using the survival R package (v3.5-7). Kaplan-Meier curves with log-rank tests were generated using the survminer package (v0.4.9). Model discrimination was assessed via Harrell’s concordance index (C-index) calculated through bootstrap validation (1,000 resamples). Predictive performance was further evaluated using receiver operating characteristic (ROC) curves with area under the curve (AUC) quantification, implemented through timeROC package. Continuous variable distributions were visualized via violin plots created with ggplot2, incorporating Wilcoxon rank-sum tests for between-group comparisons. Subgroup analyses were performed through stratified Cox models, with results presented as forest plots generated using the forestploter and jstable packages. All tests were two-sided with statistical significance defined as p < 0.05. This integrated analytical workflow leveraged Python (v3.9.16) for data preprocessing and machine learning implementations, and R (v4.2.3) for survival analyses and advanced statistical visualizations.

## 3 Results

### 3.1 Patient characteristics

**Figure 1:**
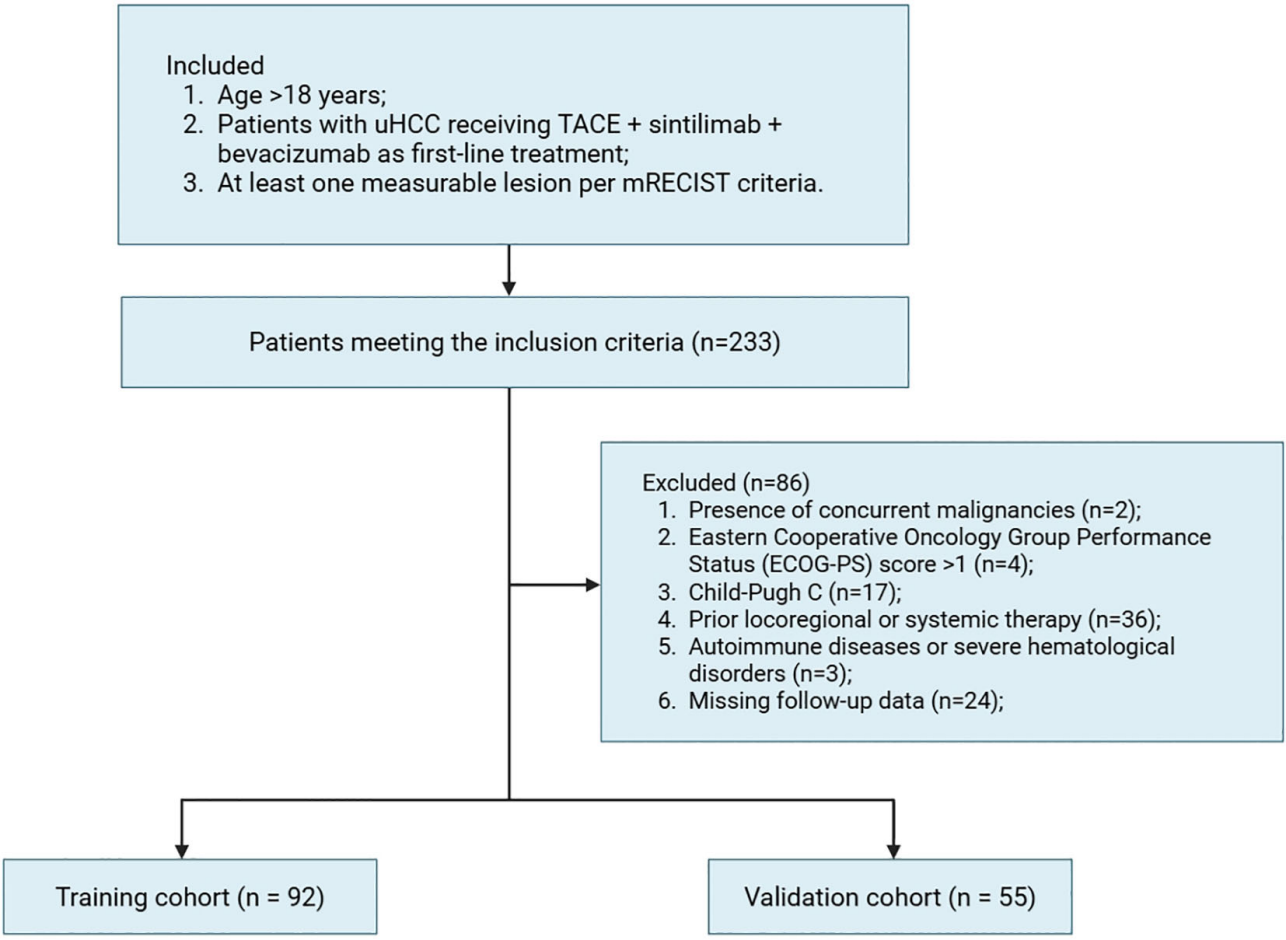
Flowchart of the study cohorts.

Among all 233 initially screened uHCC patients, 147 met eligibility criteria and were allocated to the training (n = 92) validation (n = 55) cohorts as shown in the **Figure 1**. Baseline characteristics were comparable between groups (**Table 1**). Most patients were male (~80%) and younger than 65 years (~70%), with viral hepatitis as the predominant etiology (88.0% vs. 74.5%). The majority had ECOG-PS 0–1 and Child-Pugh A liver function. Cirrhosis (75.0% vs. 65.5%) and portal vein tumor thrombosis (40.2% vs. 43.6%) were common. Approximately half had AFP >400 ng/mL, and most presented with multiple tumors, with tumor size >10 cm observed in 31.5% and 49.1% of patients. Treatment responses were similar, with ORR of 62.0% vs. 60.0% and DCR of 83.7% vs. 87.3%, confirming overall cohort comparability.

**Table 1:** Baseline characteristics of the training and validation cohort.

Parameter	Training cohort	Validation cohort	*P* value
	(n = 92)	(n = 55)	
Gender Female Male	19 (20.7%) 73 (79.3%)	10 (18.2%) 45 (81.8%)	0.881
Age (years) ≤ 65 > 65	64 (69.6%) 28 (30.4%)	41 (74.5%) 14 (25.5%)	0.687
Family History No Yes	87 (94.6%) 5 (5.4%)	52 (94.5%) 3 (5.5%)	1.0
Smoking NO Yes	69 (75.0%) 23 (25.0%)	34 (61.8%) 21 (38.2%)	0.133
Drinking No Yes	73 (79.3%) 19 (20.7%)	37 (67.3%) 18 (32.7%)	0.151
Etiology Viral Non-viral	81 (88.0%) 11 (12.0%)	41 (74.5%) 14 (25.5%)	0.060
ECOG-PS 0 1-2	57 (62.0%) 35 (38.0%)	36 (65.5%) 19 (34.5%)	0.803
Child-Pugh stage A B	76 (82.6%) 16 (17.4%)	45 (81.8%) 10 (18.2%)	1.0
BCLC stage 0 A B C	1 (1.1%) 15 (16.3%) 32 (34.8%) 44 (47.8%)	1 (1.8%) 10 (18.1%) 15 (27.3%) 29 (52.7%)	0.806
Cirrhosis: No Yes	23 (25.0%) 69 (75.0%)	19 (34.5%) 36 (65.5%)	0.293
PVTT: No Yes	55 (59.8%) 37 (40.2%)	31 (56.4%) 24 (43.6%)	0.815
AFP (ng/mL) ≤ 400 > 400	48 (52.2%) 44 (47.8%)	23 (41.8%) 32 (58.2%)	0.296
Tumor Number Solitary Multiple	29 (31.5%) 63 (68.5%)	17 (30.9%) 38 (69.1%)	1.0
Tumor Size (cm) ≤ 5 5-10	16 (17.4%) 47 (51.1%)	6 (10.9%) 22 (40.0%)	0.097
>10	29 (31.5%)	27 (49.1%)	
AST (U/L) > 40 ≤ 40	38 (41.3%) 54 (58.7%)	17 (30.9%) 38 (69.1%)	0.278
FCR > 0.8 ≤ 0.8	57 (62.0%) 35 (38.0%)	31 (56.4%) 24 (43.6%)	0.620
ORR No Yes	35 (38.0%) 57 (62.0%)	22 (40.0%) 33 (60.0%)	0.951
DCR No Yes	15 (16.3%) 77 (83.7%)	7 (12.7%) 48 (87.3%)	0.727

ECOG-PS, Eastern Cooperative Oncology Group Performance Status; BCLC, Barcelona Clinic Liver Cancer; PVTT, portal vein tumor thrombus; AFP, alpha-fetoprotein; AST, aspartate aminotransferase; FCR, fibrin degradation products to cholinesterase ratio*1000; ORR, objective response rate; DCR, disease control rate.

### 3.2 Treatment response and safety assessment

The objective response rates (ORR) were 62.0% and 60.0%, while the disease control rates (DCR) were 83.7% and 87.3% in the training and validation cohorts, respectively. Median OS were 19.0 and 18.0 months in the training and validation cohorts (**Supplementary Figures S1** and **S2**), while median PFS were both 15 months in the training and validation cohorts (**Supplementary Figures S3** and **S4**).

Among patients treated with the sintilimab and bevacizumab combination, treatment-related adverse events (TRAEs) were frequent but largely manageable (**Supplementary Table S1**). In the training cohort, 84.8% of patients experienced any-grade TRAEs, with 60.9% being Grade 1-2, 19.6% Grade 3, and 4.3% Grade 4. Similarly, in the validation cohort, 83.6% reported any-grade TRAEs, including 60.0% Grade 1-2, 18.2% Grade 3, and 5.5% Grade 4. The most common any-grade adverse events across both cohorts were: Abnormal liver function (62.0% training, 61.8% validation) Fever (30.4% training, 29.1% validation) Hypertension (27.2% training, 25.5% validation) Fatigue (23.9% training, 23.6% validation). Most events were mild to moderate (Grade 1-2). Severe events (Grade ≥ 3) occurred in 19.6% (training) and 18.2% (validation) for Grade 3, and 4.3% (training) and 5.5% (validation) for Grade 4. No treatment-related deaths were reported, and most high-grade events were managed through dose adjustments or temporary therapy discontinuation.

### 3.3 Independent prognostic factors and modeling of FAAP scoring system

**Figure 2:**
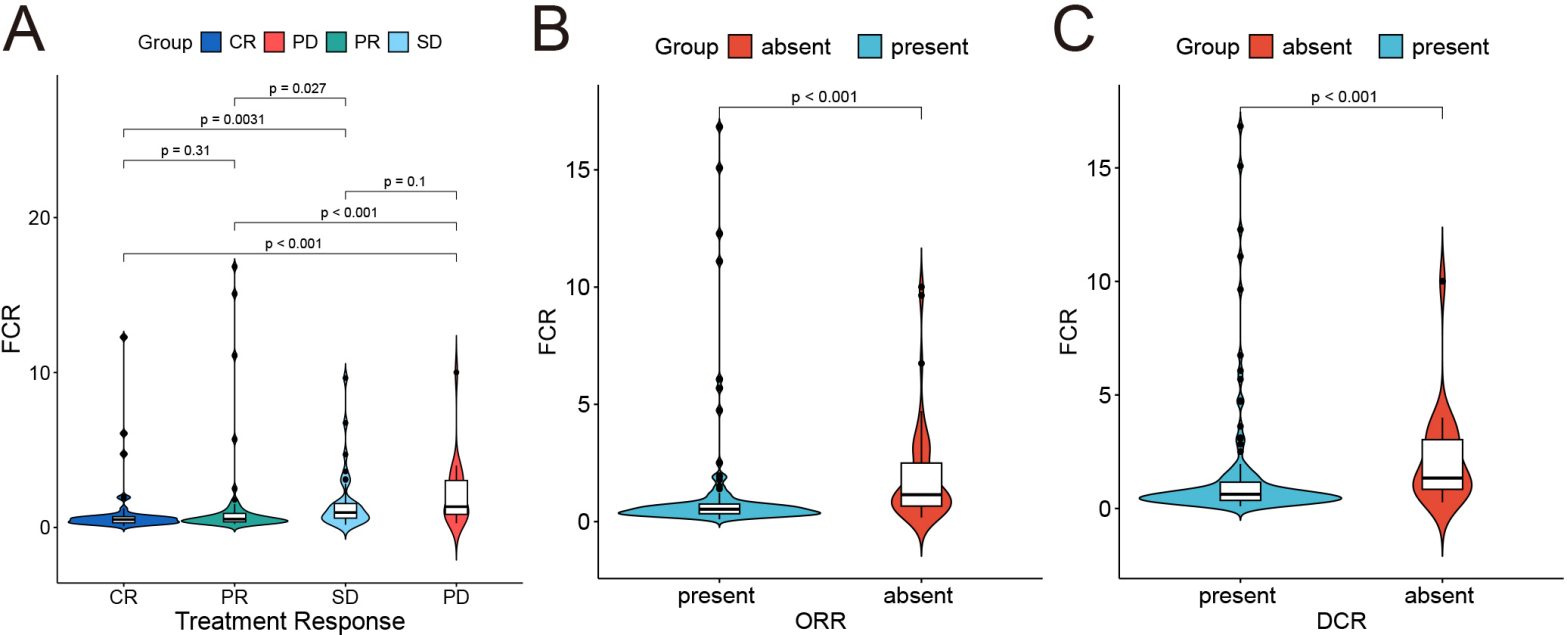
Correlation between fibrin degradation product-to-cholinesterase ratio*1000 (FCR) and treatment response. **(A)** Distribution of FCR across treatment response groups. **(B, C)** Binary classification of objective response rate (ORR) and DCR status (absent vs. present) and its association with FCR.

Fibrin degradation product-to-cholinesterase ratio (FCR) was determined as (FDP [mg/L]/CHE [U/L]) *1000, with an optimal cutoff value of 0.8 determined by X-tile analysis. Elevated FCR (> 0.8) demonstrated significant correlations with poor therapeutic outcomes (**Figure 2**): patients with high FCR showed reduced objective response rates (ORR: 28.6% vs. 63.2%; p < 0.001) and disease control rates (DCR: 64.3% vs. 89.5%; p = 0.003) compared to low-FCR patients. Violin plots revealed distinct FCR distributions across response categories (CR/PR/SD/PD), with progressive disease (PD) cases exhibiting median FCR values 2.3-fold higher than complete responders (CR) (p < 0.001).

**Figure 3:**
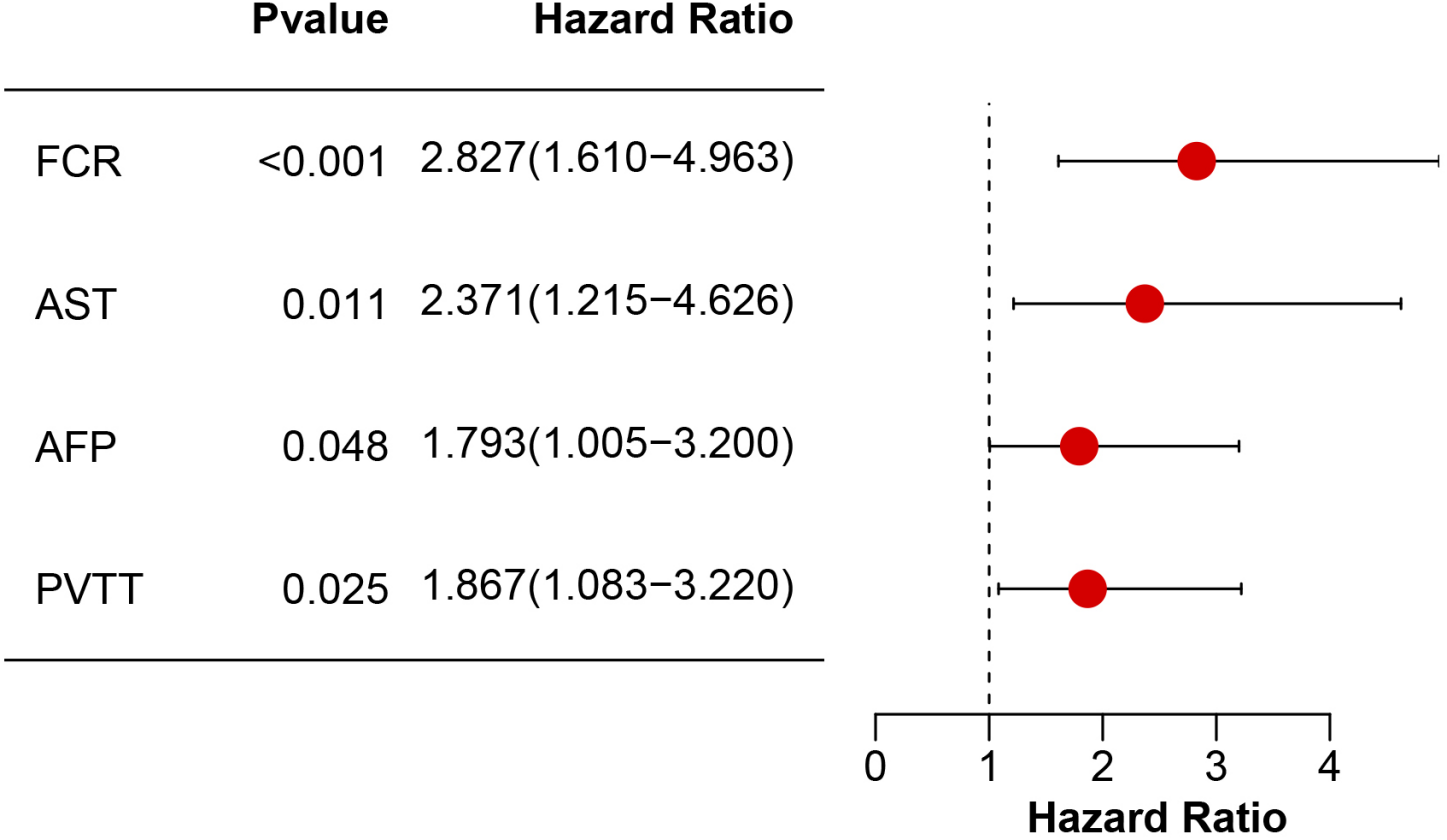
Multivariable Cox proportional hazards model analysis of prognostic factors. Hazard ratios (HR) with 95% confidence intervals (CI) and corresponding p-values are shown for FCR, AST, AFP, and PVTT.

Univariable Cox regression identified four significant predictors of OS (**Table 2**): fibrin degradation product-to-cholinesterase ratio (FCR > 0.8: HR = 3.24, 95% CI: 1.89–5.57; p < 0.001), aspartate aminotransferase (AST > 40 U/L: HR = 3.64, 95% CI: 1.94–6.84; p < 0.001), alpha-fetoprotein (AFP > 400 ng/mL: HR = 2.42, 95% CI: 1.40–4.21; p = 0.002), and portal vein tumor thrombosis (PVTT: HR = 1.97, 95% CI: 1.16–3.37; p = 0.013). Variables including age, gender, drinking, ALBI grade, ALT, cirrhosis status, tumor number and tumor size showed no significant associations (p≥0.05). In the multivariable model (**Figure 3**), FCR retained the strongest predictive value (HR = 2.83, 95% CI: 1.61–4.96; p < 0.001), followed by AST (HR = 2.37, 95% CI: 1.22–4.63; p = 0.011), PVTT (HR = 1.87, 95% CI: 1.08–3.22; p = 0.025), and AFP (HR = 1.79, 95% CI: 1.01–3.20; p = 0.048).

**Table 2:** Univariable Cox regression analyses of clinical variables for the training cohort.

Variable	Univariate analysis		
	HR	95% CI	P value
Age (≤ 65 vs. > 65)	0.68	0.36-1.26	0.218
Gender (male *vs.* female)	0.85	0.44-1.61	0.619
Drinking (present *vs.* absent)	0.98	0.49-1.94	0.948
FCR (> 0.8 *vs.* ≤ 0.8)	3.24	1.89-5.57	**< 0.001**
AST (> 40 *vs.* ≤ 40) (U/L)	3.64	1.94-6.84	**< 0.001**
ALT (> 38 *vs.* ≤ 38) (U/L)	1.57	0.92-2.70	0.094
Cirrhosis (present *vs.* absent)	1.83	0.91-3.64	0.085
AFP (> 400 *vs.* ≤ 400) (ng/mL)	2.42	1.40-4.21	**0.002**
ALB (≥ 35 *vs.* < 35) (g/L)	1.62	0.89-2.94	0.113
HBV (present *vs.* absent)	1.04	0.51-2.12	0.919
ALBI grade* (2 and 3 *vs.*1)	1.19	0.73-1.93	0.486
PVTT (present *vs.* absent)	1.97	1.16-3.37	**0.013**
Tumor size (> 10 *vs.* ≤ 10) (cm)	1.30	0.86-1.96	0.219
Extrahepatic metastasis (present *vs.* absent)	1.19	0.63-2.23	0.577
Tumor number (multiple *vs.* solitary)	1.03	0.57-1.85	0.901

*ALBI score= (log 10 TBil [μmol/L] × 0.66) + (albumin [g/L] × − 0.085); ALBI grade 1: ALBI.

score ≤ − 2.60; ALBI grade 2: ALBI score − 2.60 to − 1.39; ALBI grade 3: ALBI score ≥ 1.39.

FCR, fibrin degradation products to cholinesterase ratio*1000; HR, hazard ratio; CI, confidence interval; AST, aspartate aminotransferase; ALT, alanine aminotransferase; AFP, alpha-fetoprotein; ALB, albumin; HBV, hepatitis B virus; PVTT, portal vein tumor thrombus.Bold values indicate statistical significance (p < 0.05).

The prognostic model was designated as the FAAP score (**F**CR, **A**ST, **A**FP, **P**VTT). Which is calculated as: FAAP Score = 0.891 × FCR (0 or 1) + 0.746 × AST (0 or 1) + 0.526 × AFP (0 or 1) + 0.528 × PVTT (0 or 1), where each variable is dichotomized using predefined thresholds (FCR > 0.8, AST > 40 U/L, AFP > 400 ng/mL, PVTT presence).

### 3.4 Performance of FAAP scoring system

**Figure 4:**
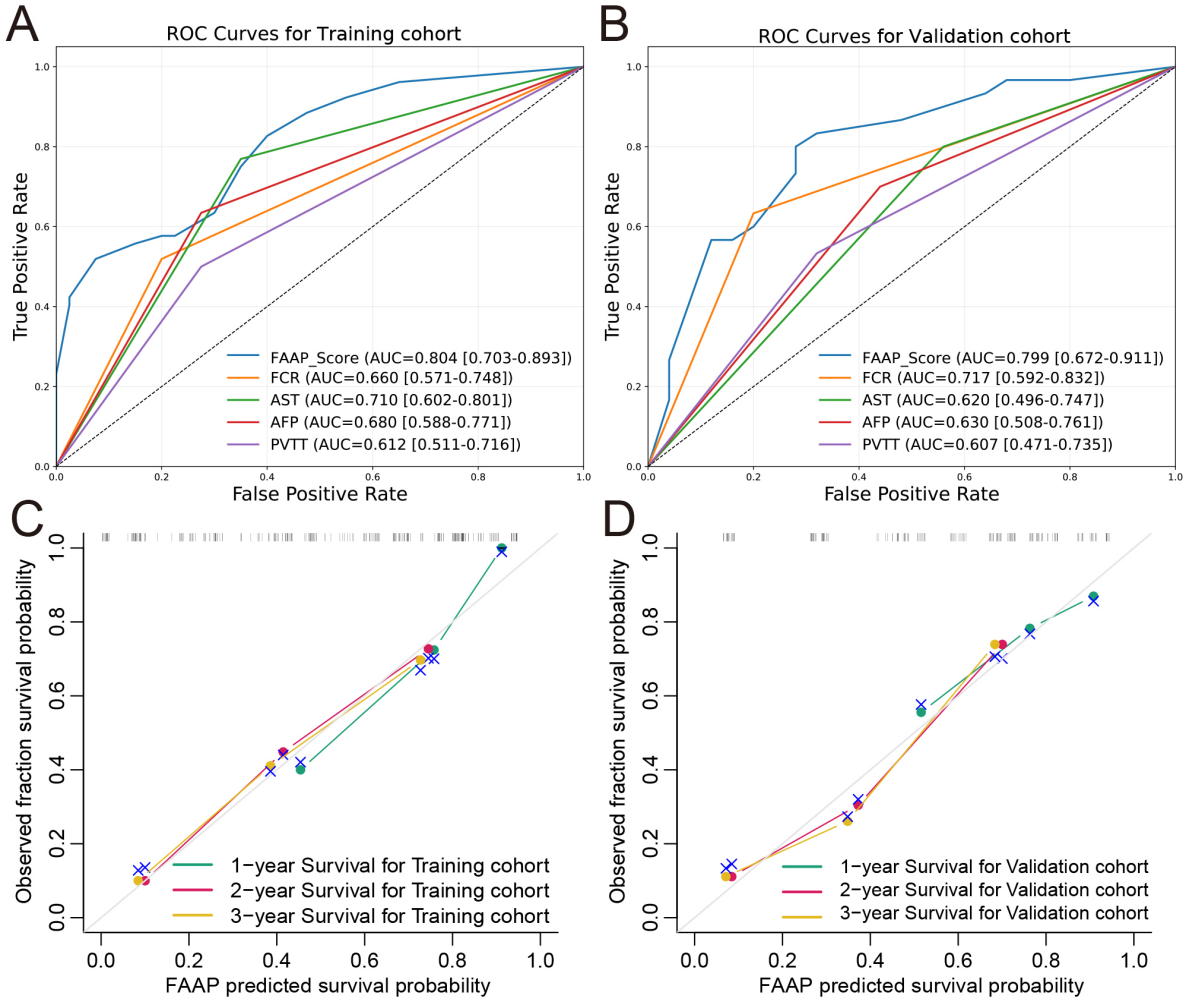
Predictive performance and calibration performance of the FAAP prognostic model. Receiver operating characteristic (ROC) curves for the predictive performance of the FAAP score in the training **(A)** and validation **(B)** cohorts. Observed versus predicted survival probabilities for one-year, two-year, and three-year survival in the training **(C)** and validation **(D)** cohorts, respectively.

The FAAP scoring system demonstrated favorable discriminative performance in both training and validation cohorts. As shown in **Figures 4A, B**, the FAAP score showed higher AUC values compared with established clinical parameters in the training cohort (AUC = 0.804, 95% CI: 0.703-0.893; FCR 0.660, AST 0.710, AFP 0.680, PVTT 0.612), while showing comparable accuracy in the validation cohort (AUC = 0.799, 95% CI: 0.672-0.911; FCR 0.717, AST 0.620, AFP 0.630, PVTT 0.607). The calibration curves for the training (**Figure 4C**) and validation (**Figure 4D**) cohorts demonstrated excellent agreement between the predicted survival probabilities and the observed survival fractions. The plots for 1-, 2-, and 3-year survival closely followed the diagonal reference line, indicating well-calibrated models without significant over- or under-estimation. These findings were supported by C-index analyses, with C-index values of 0.754 (95% CI: 0.691-0.813) and 0.722 (95% CI: 0.615-0.825) observed in the training and validation cohorts, respectively.

### 3.5 Survival analysis of FAAP scoring system

**Figure 5:**
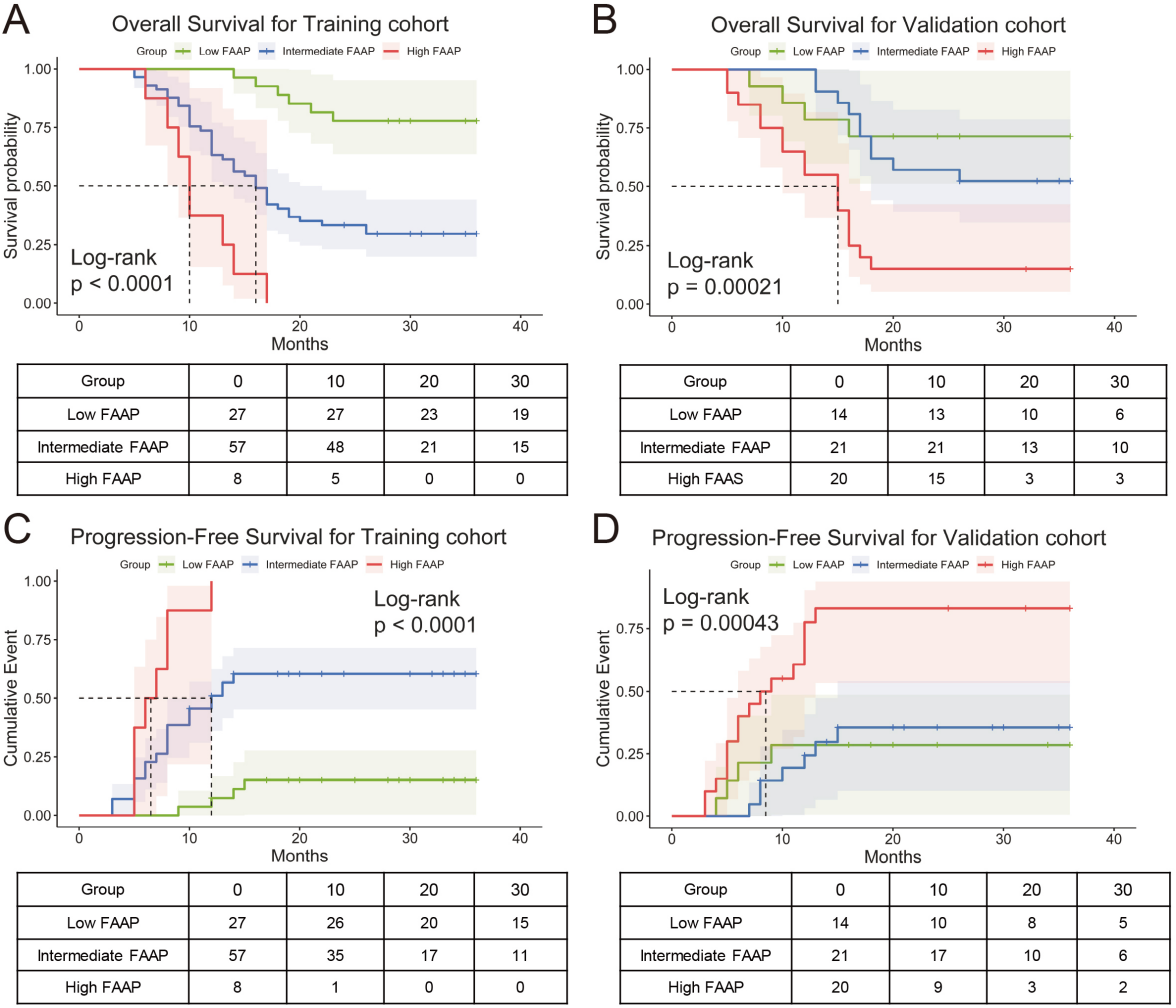
Prognostic stratification by FAAP score in training and validation cohorts. **(A)** Kaplan-Meier curves for overall survival (OS) in the training cohort, stratified into low, intermediate, and high FAAP score groups (Log-rank p < 0.0001). **(B)** Survival probability curves in validation cohort across FAAP subgroups (Log-rank p = 0.00021). **(C, D)** Progression-free survival (PFS) analysis for the training cohort (Log-rank p < 0.0001) and validation cohort (Log-rank p = 0.00043), respectively.

The FAAP score was stratified into three different risk group using X-tile: low (FAAP < 0.7), intermediate (0.7 ≤ FAAP < 2.2), and high (FAAP ≥ 2.2). Kaplan–Meier survival analysis demonstrated significant differences in OS among patients classified into low, intermediate, and high FAAP groups in both cohorts (**Figures 5A, B**). The log-rank tests confirmed that these differences were statistically significant, reflecting the strong prognostic value of the FAAP score. In addition, progression-free survival (PFS) analysis showed that patients with higher FAAP scores had significantly shorter PFS compared to those with lower scores, as presented in **Figures 5C, D**. These findings collectively suggest that the FAAP scoring system effectively stratifies patients by risk and may serve as a reliable predictor of both overall and progression-free survival in HCC.

### 3.6 Subgroup analysis of FAAP prognostic efficacy

**Figure 6:**
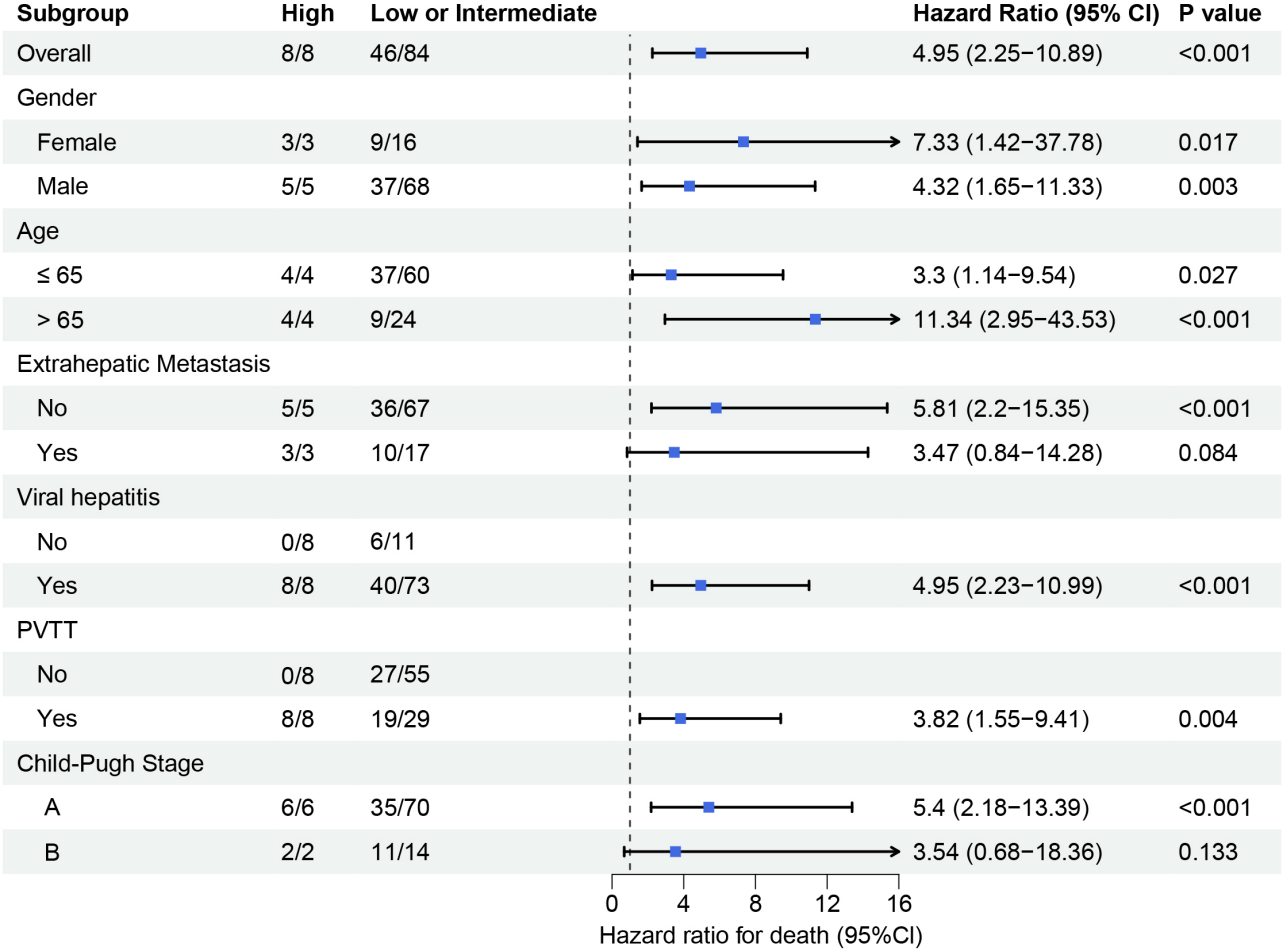
Subgroup analysis of survival outcomes stratified by clinical and pathological characteristics. Forest plot displays hazard ratios (HR) with 95% confidence intervals (CIs) for mortality risk across different subgroups. High FAAP score subgroups (vs. low/intermediate) consistently exhibited elevated mortality risk.

Subgroup analysis demonstrated consistent prognostic performance of the FAAP score across clinically relevant subgroups as shown in **Figure 6**. High-risk patients (FAAP ≥2.2) exhibited an increased mortality risk compared to low/intermediate-risk patients (95% CI: 2.25–10.89; p < 0.001). The association remained significant in male (HR = 4.32, p = 0.003) and female subgroups (HR = 7.33, p = 0.017), with amplified effects observed in elderly patients (> 65 years: HR = 11.34, p < 0.001). Notably, the FAAP score retained predictive validity regardless of portal vein tumor thrombosis status (PVTT-positive: HR = 3.82, p = 0.004) and Child-Pugh classification (Stage A: HR = 5.40, p < 0.001). Subgroup heterogeneity emerged in extrahepatic metastasis cohorts (metastasis-positive: HR = 3.47, p = 0.084), potentially reflecting limited sample size rather than biological variation. Viral hepatitis-negative patients showed no calculable risk due to complete early mortality (8/8 deaths) in the high-FAAP group.

## 4 Discussion

This study established and validated the FAAP scoring system, a novel prognostic model integrating fibrin degradation product-to-cholinesterase ratio*1000 (FCR), aspartate aminotransferase (AST), alpha-fetoprotein (AFP), and portal vein tumor thrombosis (PVTT) to predict survival outcomes in uHCC patients receiving triple therapy with TACE, sintilimab and bevacizumab. The FAAP score demonstrated robust discriminative performance for stratifying patients into distinct risk groups, with significant prognostic relevance for both OS and PFS. By addressing the unmet need for predictive tools in this therapeutic context, our model provides clinicians with a practical framework to optimize treatment selection and prognosis evaluation.

Previous studies have reported that the combination of TACE and anti-PD-1 therapy achieves an ORR of approximately ≥ 50% (23, 24). In our study, the ORR was 61.96% in the training cohort and 60.00% in the validation cohort, with the addition of bevacizumab further improving the ORR. This underscores the critical role of antiangiogenic therapy in uHCC treatment. According to recent clinical trials or multicenter studies, anti-PD-1/PD-L1 plus anti-VEGF yields an ORR of 25%-31% (10, 25, 26). On this basis, our study incorporated TACE, significantly enhancing the ORR in uHCC patients and demonstrating the feasibility of this regimen. Additionally, in our study, the median OS and PFS in the training cohort were 19.0 and 15.0 months, respectively. Given the complexity of TACE procedures, as well as differences in follow-up duration, baseline characteristics, and sample size compared to the CHANCE2201 study, these outcomes are acceptable (17). Although clinical trial cohorts are often highly selected, our findings remain largely consistent with previous studies (6, 27). These results further indicate that TACE may provide complementary benefits to the combined therapy of sintilimab and bevacizumab.

When further analyzing treatment efficacy, safety and tolerability should also be considered. Although the combination therapy demonstrated a significant improvement in ORR, the associated adverse events and their management remain crucial. In our study, most patients tolerated the combination of bevacizumab, TACE, and anti-PD-1 therapy, with an adverse event profile consistent with previous reports (28, 29). This suggests that the regimen achieves a reasonable balance between efficacy and tolerability. Moreover, accumulating long-term follow-up data will allow for a more comprehensive assessment of the durability and potential survival benefits of this strategy. Future studies could explore response variations among patients with different baseline characteristics to optimize individualized treatment approaches and further improve outcomes in uHCC.

The FAAP model developed in this study integrates four variables: FCR (fibrinogen degradation product/cholinesterase ratio*1000), AST, AFP, and PVTT. First of all, FCR is an innovative composite biomarker, which simultaneously reflects coagulation-fibrinolysis activation (elevated FDP) and impaired hepatic synthetic function (decreased cholinesterase). Prior studies suggest that elevated FDP is associated with HCC and other malignancies, serving as a diagnostic biomarker to differentiate malignant from non-malignant ascites and correlating with liver dysfunction or cirrhosis (30–32). Meanwhile, decreased cholinesterase indicates compromised liver reservation function. FCR may more sensitively capture the synergistic effect between abnormal coagulation and liver dysfunction, However, the dual-dimensional characteristics of coagulation and metabolism of FCR are unique, even though this idea is similar to the recently proposed composite indicators such as systemic immune inflammation index (SII) (33). Second, AST, AFP, and PVTT are validated prognostic factors, but their weights in the context of combined treatment are worthy of exploration. AST is a marker of liver injury, which correlates with post-resection HCC recurrence, and its inclusion reflects the impact of local treatment-related hepatotoxicity on survival (34). AFP serves as a tumor biological marker, retains prognostic value in the era of immunotherapy, consistent with subgroup analyses from trials such as IMbrave150 (7). PVTT is a vascular invasion indicator, which directly affects TACE efficacy and systemic treatment response, with its prognostic significance repeatedly validated in studies combining TACE with immunotherapy (35, 36). Compared to other models (e.g., ALBI focusing on liver function, mRECIST emphasizing tumor burden), FAAP more precisely evaluates local-systemic interactions by incorporating PVTT. The similarities and differences with previous models deserve attention: (1) BCLC and CNLC remain the primary staging system for determining treatment methods, while FAAP serves as a complementary, biomarker-based prognostic tool that stratifies risk among patients managed with a similar treatment; (2) Unlike immunotherapy-specific models, Prognostic Nutritional Index (PNI) or PD-L1 expression, FAAP excludes direct immune parameters but may indirectly reflect tumor microenvironment. Additionally, while similar combination therapy studies (LEAP-012) often include ECOG performance status or tumor burden metrics, FAAP prioritizes liver function and tumor biology, highlighting divergent core prognostic factors across treatment modalities.

Briefly, the FAAP variables might distinguish prognosis in patients receiving TACE plus anti-VEGF and PD-1 blockade. FCR integrates fibrinolysis activity and hepatic synthetic reserve, both linked to ischemia–hypoxia, vascular remodeling, and systemic therapy tolerance. AST reflects baseline hepatocellular injury, which influences both TACE-related hepatic stress and the capacity to continue systemic therapy. AFP indicates tumor burden and aggressiveness. PVTT represents macrovascular invasion that limits locoregional control and alters perfusion/immune microenvironment. Together, these dimensions provide a biologically plausible, biomarker-based prognostic portrait for patients managed with the same triple-therapy rationale, while not implying prediction of treatment benefit across different therapies.

This study has several limitations. First, although standardization of TACE procedures within a single center offers advantages, inherent variability in TACE administration remains unavoidable. Second, Given the retrospective design and modest sample size, potential selection bias is inevitable. We will first expand the sample size and continue follow up, then initiate prospective or ambispective multicenter validation to confirm generalizability and clinical utility. Third, the follow-up time limits the assessment of long-term outcomes, particularly for patients receiving combination immunotherapy. Therefore, extended observation is still required to evaluate survival benefits and delayed toxicities. Finally, the clinical utility of the FAAP scoring system remains to be defined, including how dynamic parameter changes may influence its predictive performance.

In conclusion, the FAAP scoring system effectively stratifies uHCC patients undergoing TACE-sintilimab-bevacizumab therapy into distinct prognostic groups, offering a clinically accessible tool for personalized management. Its derivation from readily available parameters enhances translational applicability, though prospective validation is essential to confirm its generalizability and refine risk-adapted therapeutic strategies.

## Data Availability

The data analyzed in this study is subject to the following licenses/restrictions:The datasets used and analyzed in the current study are available from the corresponding author upon reasonable request. Requests to access these datasets should be directed to chenhongsong2999@163.com.
