# Supplementary material for: Development and validation of the FAAP model for prognostic stratification in HCC patients treated with TACE, sintilimab plus bevacizumab: a multicenter study
Source: Front Immunol. 2025 Nov 25;16:1692632. doi: 10.3389/fimmu.2025.1692632 (PMC12685804; doi:10.3389/fimmu.2025.1692632)

Table S1. Treatment-Related Adverse Events

|  | Training Cohort: n = 92 | | | | Validation Cohort: n = 55 | | | |
| --- | --- | --- | --- | --- | --- | --- | --- | --- |
| Adverse Events | **Any Grade** | **Grade 1-2** | **Grade 3** | **Grade 4** | **Any Grade** | **Grade 1-2** | **Grade 3** | **Grade 4** |
| Total | 78 (84.8%) | 56 (60.9%) | 18 (19.6%) | 4 (4.3%) | 46 (83.6%) | 33 (60.0%) | 10 (18.2%) | 3 (5.5%) |
| Fatigue | 22 (23.9%) | 18 (19.6%) | 4 (4.3%) | 0 | 13 (23.6%) | 11 (20.0%) | 2 (3.6%) | 0 |
| Fever | 28 (30.4%) | 27 (29.3%) | 1 (1.1%) | 0 | 16 (29.1%) | 15 (27.3%) | 1 (1.8%) | 0 |
| Hypertension | 25 (27.2%) | 20 (21.7%) | 5 (5.4%) | 0 | 14 (25.5%) | 11 (20.0%) | 3 (5.5%) | 0 |
| Abnormal liver function | 57 (62.0%) | 47 (51.1%) | 9 (9.8%) | 1 (1.1%) | 34 (61.8%) | 28 (50.9%) | 5 (9.1%) | 1 (1.8%) |
| Nausea | 28 (30.4%) | 22 (23.9%) | 6 (6.5%) | 0 | 16 (29.1%) | 14 (25.5%) | 2(3.6%) | 0 |
| Diarrhea | 11 (12.0%) | 11 (12.0%) | 0 | 0 | 7 (12.7%) | 7 (12.7%) | 0 | 0 |
| Proteinuria | 10 (10.9%) | 7 (7.6%) | 3 (3.3%) | 0 | 6 (10.9%) | 4 (7.3%) | 2 (3.6%) | 0 |
| Rash | 9 (9.8%) | 7 (7.6%) | 2 (2.2%) | 0 | 5 (9.1%) | 4 (7.3%) | 1 (1.8%) | 0 |
| Thrombocytopenia | 13 (14.1%) | 13 (14.1%) | 0 | 0 | 8 (14.5%) | 8 (14.5%) | 0 | 0 |
| Decreased appetite | 10 (10.9%) | 8 (8.7%) | 2 (2.2%) | 0 | 6 (10.9%) | 5 (9.1%) | 1 (1.8%) | 0 |
| Abdominal pain | 8 (8.7%) | 7 (7.6%) | 1 (1.1%) | 0 | 5 (9.1%) | 4 (7.3%) | 1 (1.8%) | 0 |

Figure S1. Overall survival of all patients in the training cohort.


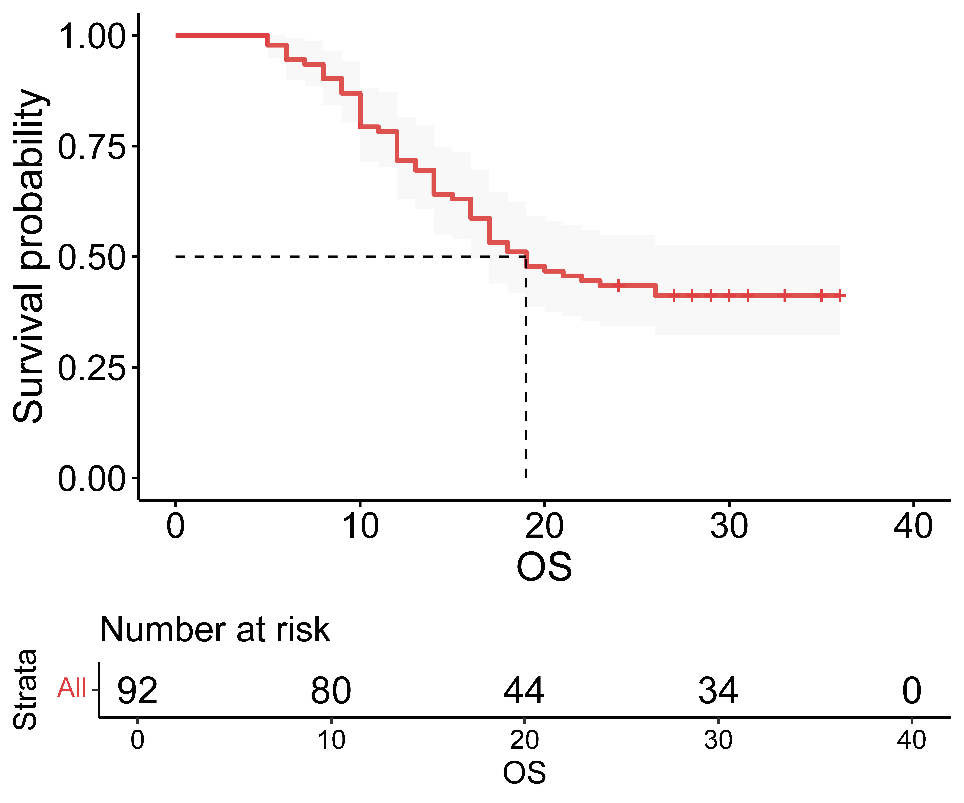


Figure S2. Progression-free survival of all patients in the training cohort.


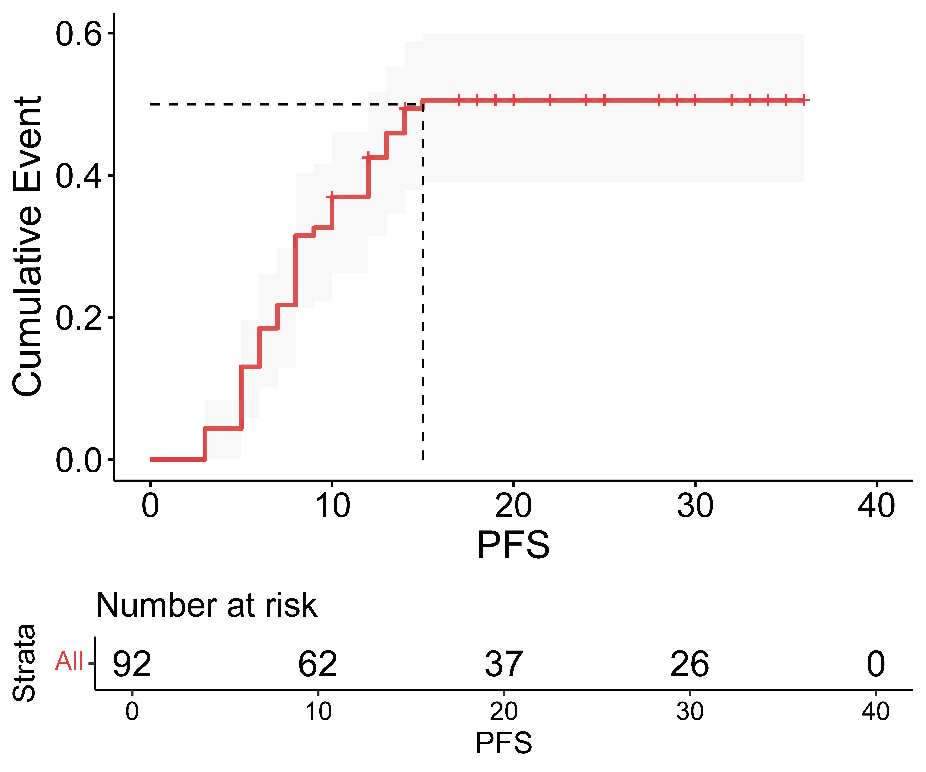


Figure S3. Overall survival of all patients in the validation cohort.


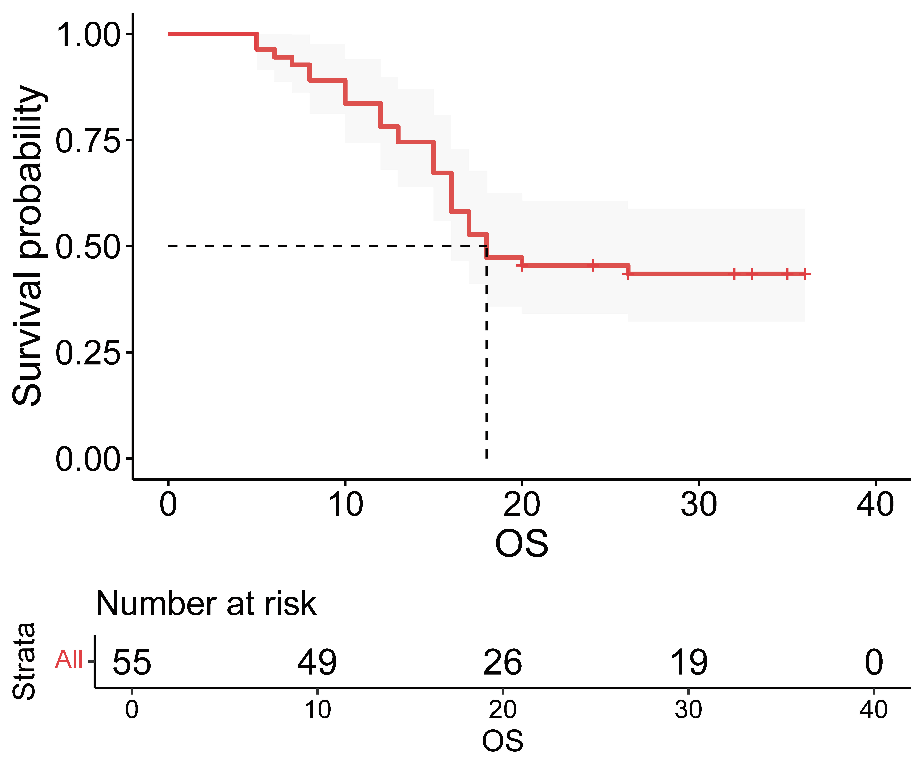


Figure S4. Progression-free survival of all patients in the validation cohort.


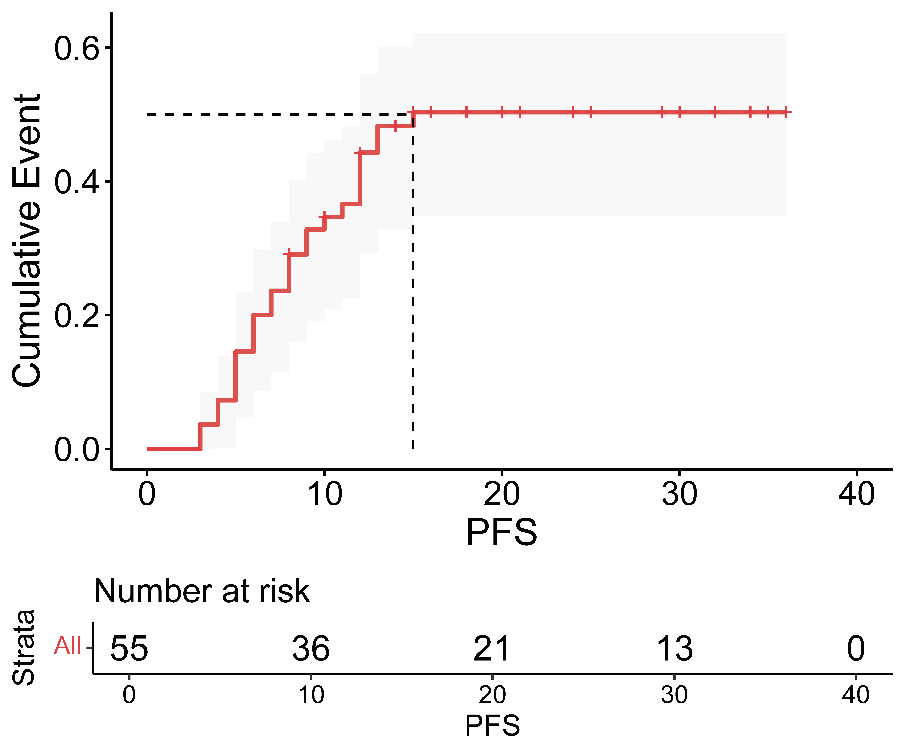


Figure S5. Nomogram of FAAP scoring system.


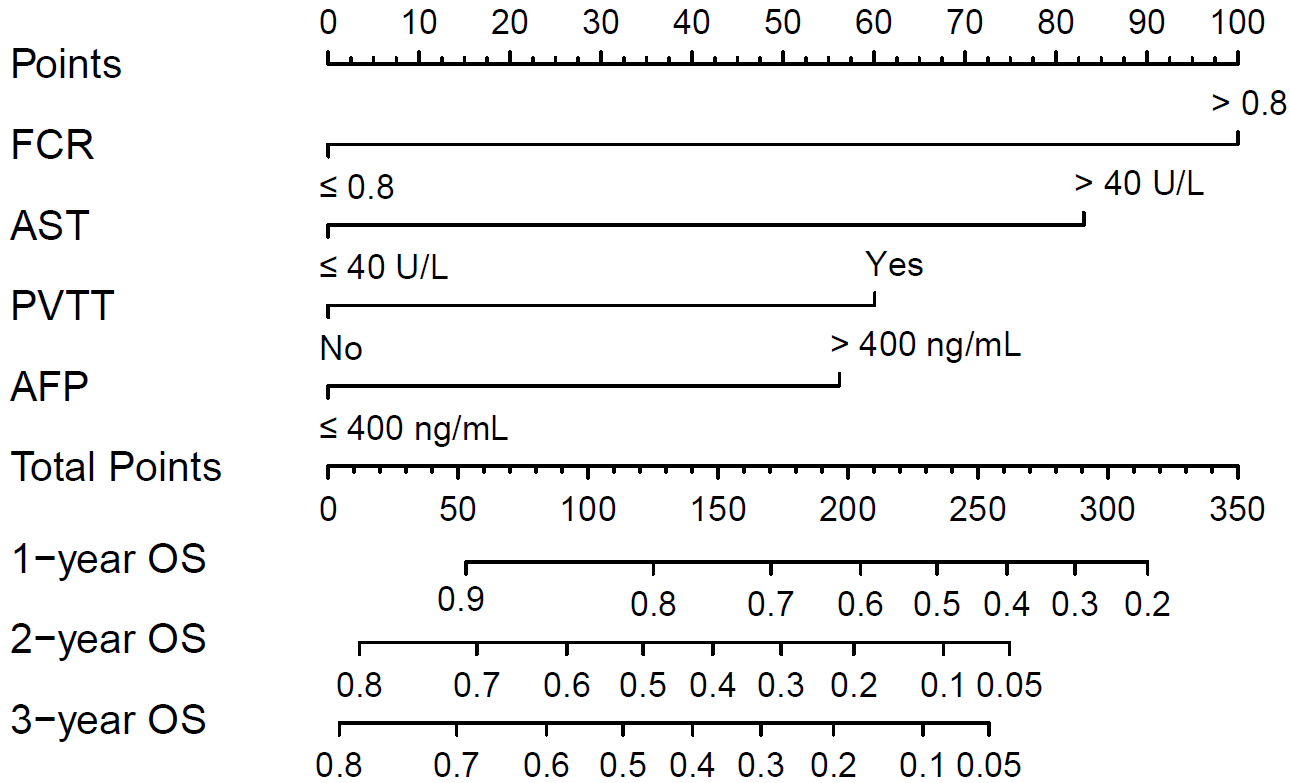

Supplement: Supplementary file 1 [file DataSheet1.docx]
